# Supplementary material for: Tumor derived UBR5 promotes ovarian cancer growth and metastasis through inducing immunosuppressive macrophages
Source: Nat Commun. 2020 Dec 8;11:6298. doi: 10.1038/s41467-020-20140-0 (PMC7722725; doi:10.1038/s41467-020-20140-0)
Supplement: Supplementary file 1 — Supplementary information [file 41467_2020_20140_MOESM1_ESM.pdf]

Supplementary Information

Tumor derived UBR5 promotes ovarian cancer growth and metastasis through inducing immunosuppressive macrophages

This file includes:

- Supplementary Figures 1-13
- Supplementary Tables 1-5

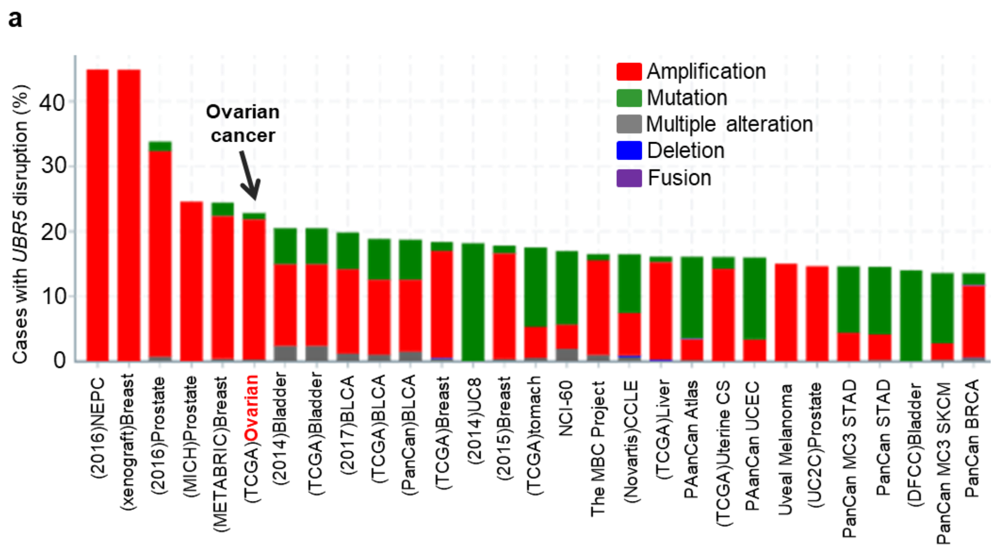

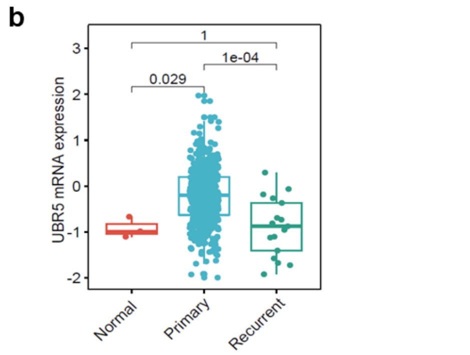

	Normal	Primary	Recurrent
Number of values	3	541	17
Minimum	-1.105	-1.997	-1.924
25% Percentile	-1.105	-0.6324	-1.493
Median	-0.9871	-0.2	-0.8771
75% Percentile	-0.6695	0.1987	-0.3176
Maximum	-0.6695	1.972	0.2939
Mean ± SEM	-0.9206 ± 0.1301	-0.2198 ± 0.02599	-0.8945 ± 0.1551

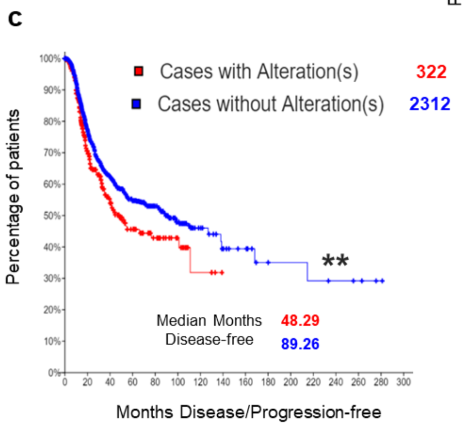

Supplementary Fig.1: Clinical observations of UBR5's involvement in ovarian cancer. Figure supplementary to main Figure 1

a A cBioPortal /TCGA-based analysis of UBR5's genomic alterations across all cancers deposited in the databases (240 studies and 78,404 cancer samples). **b** Relative mRNA expression levels of healthy individuals, patients with OC and those with recurrent OC (normal *n*=3, primary *n*=541,recurrent *n*=17 independent samples per group), The *P* values shown were calculated by unpaired two-sided Student's *t* test without adjustments. **c** Recurrence-free survival rates of ovarian cancer patients with altered UBR5 gene cases (c/BioPortal/TCGA), *P*=0.0078, log rank test[1].

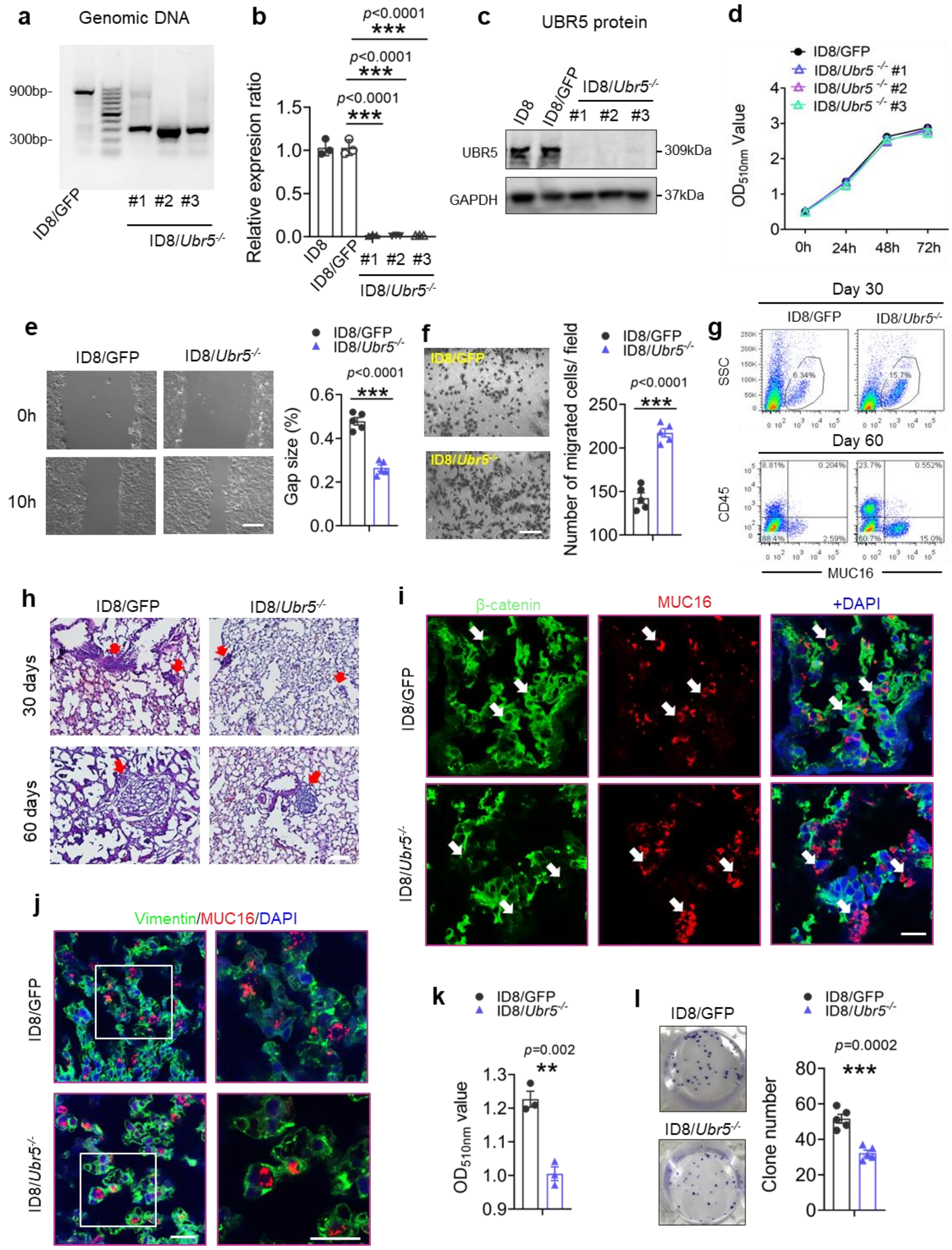

Supplementary Fig.2: Verification of ID8/*Ubr5*$^{-/-}$ monoclones *in vitro* and *in vivo*. Figure supplementary to main Figure 1

a-**c** Deletion of *Ubr5* gene in ID8-Muc16^{ecto} cells via CRISPR/Cas9 was verified at genomic DNA level (**a**), mRNA level (**b**) (*n* = 3 biologically independent samples per group), and protein level (**c**) by PCR, RT-PCR and western blot, respectively. **d** Sulforhodamine B (SRB) assay showing growth rates of ID8/GFP and ID8/*Ubr5*$^{-/-}$ monoclones (*n* = 3 biologically independent samples per group). Representative micrographs and quantitation of Transwell-migration assay (**e**) (*n* = 5 biologically

independent samples per group) or wound-healing assay (**f**) ($n = 5$ biologically independent samples per group). Scale bar: 200μm. **g**-**j** To evaluate spontaneous metastasis, 5×10^6 ID8 cells were i.v. injected to C57BL/6 female recipient mice. **g** Representative FACS images of $Muc16^+$ tumor cells in lung at indicated times after ID8 i.v. injection($n = 3$ mice per group). **h** Representative images of H&E staining of lung sections showing pulmonary metastatic nodules ($n = 3$ mice per group). Scale bar: 100μm. **i** Representative immunofluorescence (IF) images of lung sections showing β-catenin expression in $MUC16^+$ ID8/GFP cells, but not in $MUC16^+$ ID8/*Ubr5*$^{-/-}$ cells at day 30 post i.v. injection($n = 3$ mice per group). All panels are the same magnification, scale bar: 25μm. **j** Representative IF images of lung sections showing vimentin expression in $MUC16^+$ ID8 cells at day 60 post i.v. injection($n = 3$ mice per group). Scale bar: 25μm. **k** Cell adhesion assay with $1E^5$ ID8 cells ($n = 3$ biologically independent samples per group). **l** Representative images and quantified values of clonogenic assay with 50 cells /well ($n = 5$ biologically independent samples per group). All data are representative of at least two independent experiments with similar results. Data are presented as mean ± SEM, unpaired two-sided Student's *t* test with no correction for multiple comparison, $^{**}P < 0.01$, $^{***}P < 0.001$. Source data are provided as a Source Data file.

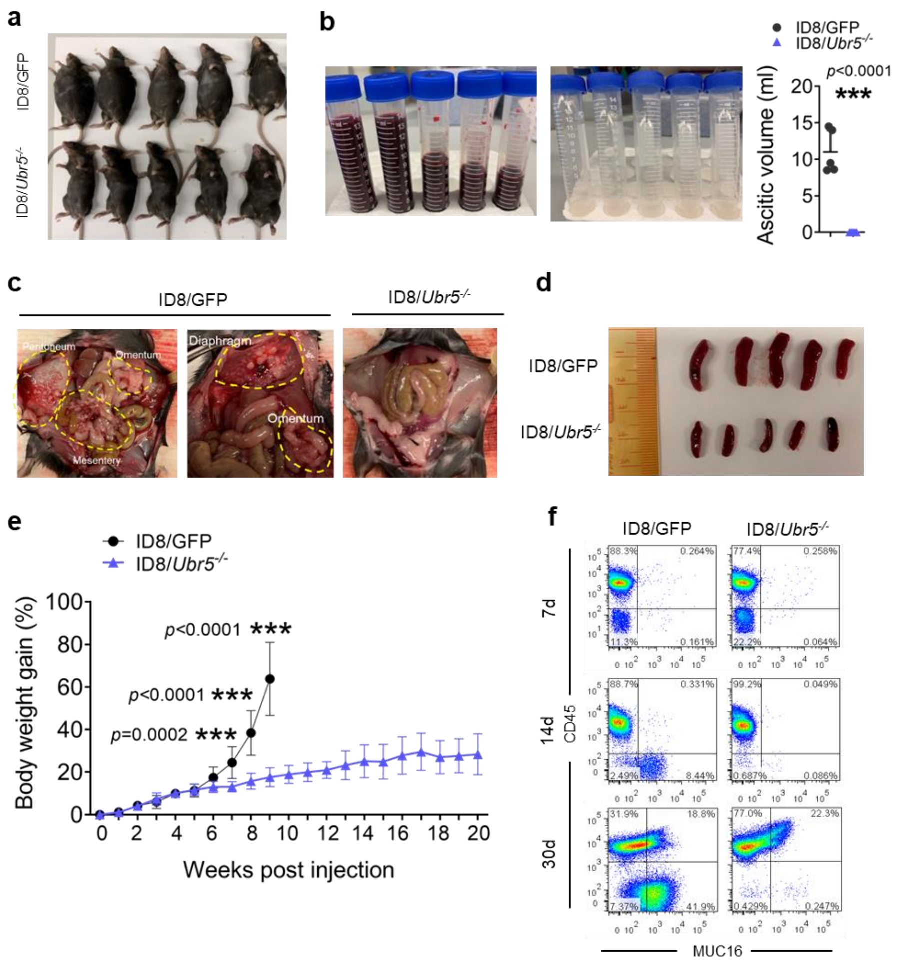

Supplementary Fig.3: Attenuated tumor growth and peritoneal implantation of ID8/*Ubr5*$^{-/-}$. Figure supplementary to main Figure 1

a-**e** An orthotopic syngeneic mouse model by implanting 1×10^{6} ID8 cells into left ovarian bursa of C57BL/6 recipient mice. Mice were euthanized at 8 weeks after tumor implantation. **a** Representative images of tumor bearing mice. **b** Representative images of malignant ascites, and the volumes of ascetic fluid were measured ($n = 5$ mice per group), data are presented as mean ± SEM, unpaired two-sided Student's *t* test. **c** Representative images of tumor implantations in omentum, peritoneum, mesentery and diaphragm. **d** Reduced splenomegaly in bearing mice. **e** Mouse body weights were monitored twice a week, and ascites generation in ID8/*Ubr5*$^{-/-}$ bearing mice expressed as percent weight gain (ID8/GFP $n = 18$, ID8/*Ubr5*$^{-/-}$ n=13 biologically independent samples per group), data are presented as mean ± SD, unpaired two-sided Student's *t* test. **f** Representative FACS images of Muc16^{+} CD45^{-} tumor cells retrieved from peritoneal ascites at indicated times after i.p. injection. All data are representative of at least two independent experiments with similar results. ***P < 0.001. Source data are provided as a Source Data file.

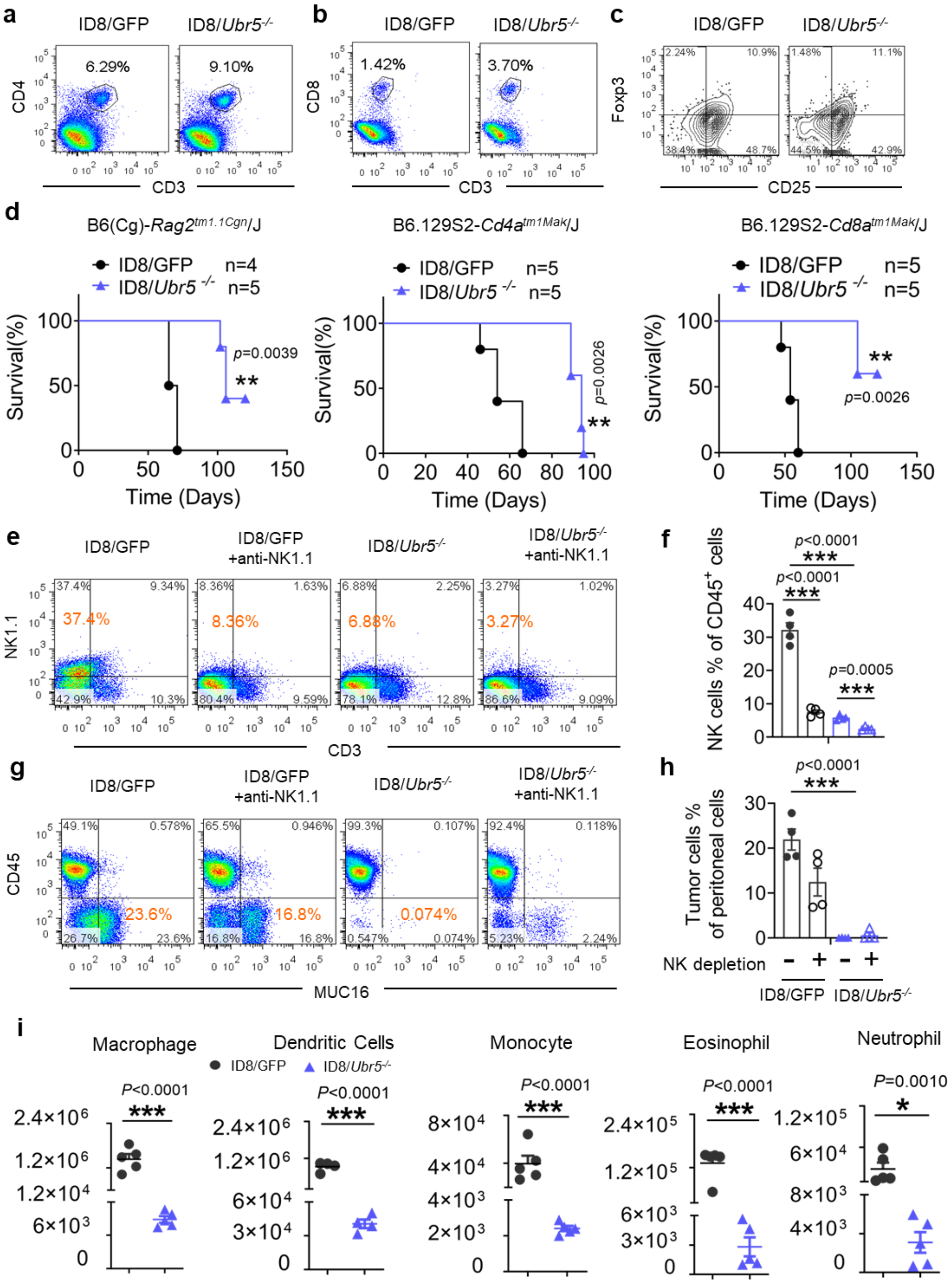

Supplementary Fig.4: Lack of adaptive immunity involvement but strong reduction of macrophages in the tumor microenvironment of ID8/*Ubr5*$^{-/-}$. Figure supplementary to main Figure 2

a-b More CD4^{+} / CD8^{+} T cells infiltrated into peritoneal cavity at 1week post tumor implantation in ID8/*Ubr5*$^{-/-}$ bearing mice. Representative FACS images of peritoneal

$CD4^+$ T cells and $CD8^+$ T cells gated on $CD45^+$ cells. **c** Representative FACS images of $Foxp3^+CD25^+$ regulatory T cells gated on $CD45^+$ $CD3^+$ $CD4^+$ cells. **d** Survival in *Rag2*$^{-/-}$, CD4-deficient and CD8-deficient mice bearing ID8 tumors (*n* =4- 5 mice per group), log rank test. Data are representative of at least two independent experiments with similar results. **e**-**f** Representative FACS images and quantification of infiltrated $CD3^-NK1.1^+$ NK cells (*n* =4 mice per group). **g**-**h** Representative FACS images and quantification of $Muc16^+$ $CD45^-$ tumor cells in peritoneal cavity (*n* =4 mice per group). **i** Reduced infiltration of myeloid cell population in ID8/*Ubr5*$^{-/-}$ bearing mice. The amount of macrophages ($F4/80^+CD11b^+$Siglec F^-Ly6C^-), Monocytes ($F4/80^+CD11b^+$ $Ly6C^+$ Siglec F^-) Eosinophils (Siglec $F^+F4/80^+$), Neutrophils ($F4/80^-$ Siglec $F^-Ly6G^+Ly6C^+$), dendritic cells ($CD11c^+MHCII^+$) in peritoneal cavity (*n* =5 mice per group). All data are representative of at least two independent experiments with similar results. Data are presented as mean ± SEM, unpaired two-sided Student's *t* test with no correction for multiple comparison,**P < 0.01, ***P < 0.001. Source data are provided as a Source Data file.

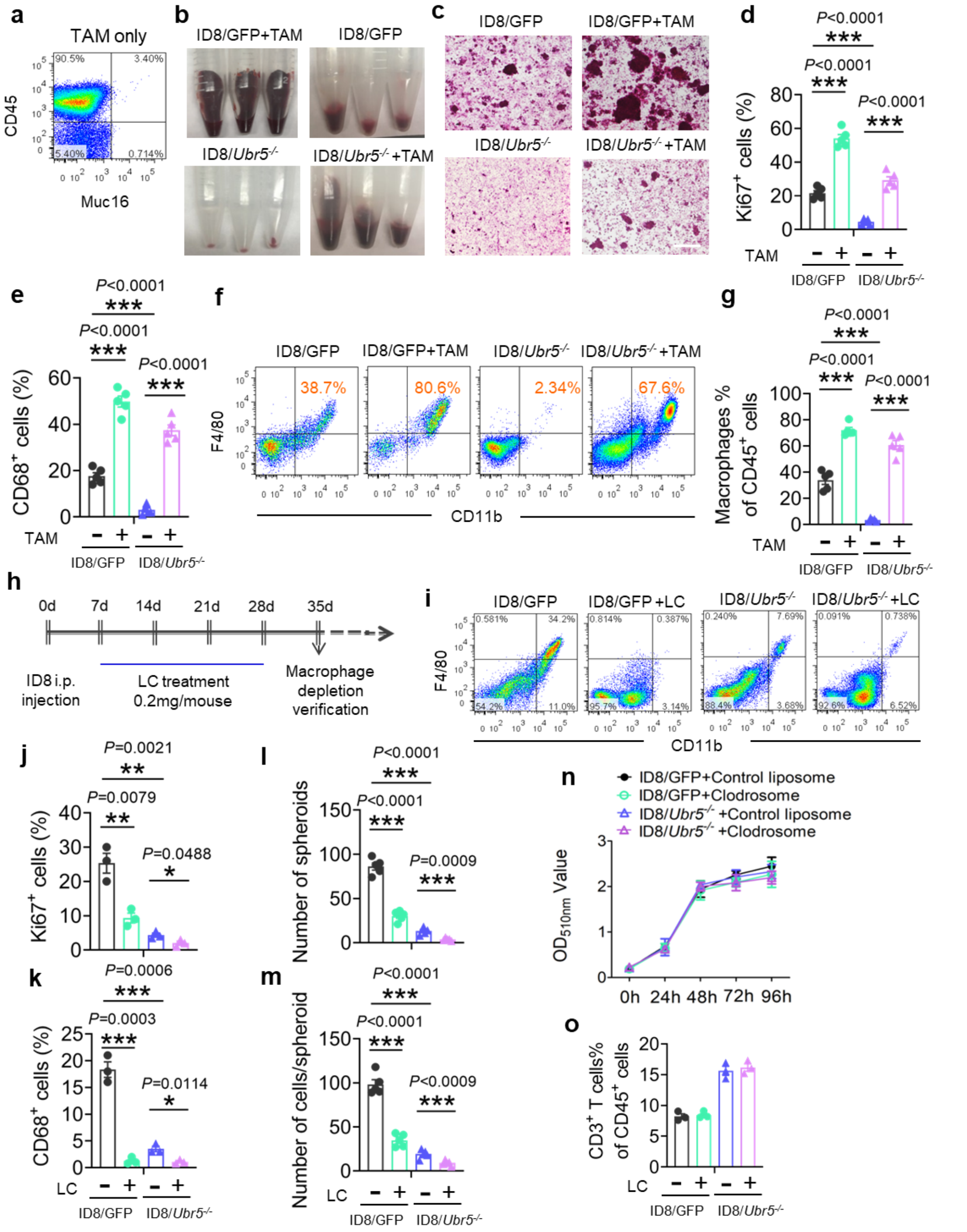

Supplementary Fig.5: Impaired macrophage recruitment caused by *Ubr5* deficiency attenuated peritoneal OC growth. Figure supplementary to main Figure 3

a Injection of TAMs alone did not produce tumors. Peritoneal cells were retrieved at day 50 post TAMs *i.p.* injection (*n* =5 mice per group). **b-g** Adoptive transfer of TAMs boosted ID8/*Ubr5*$^{-/-}$ tumor growth to control levels in mice. Peritoneal washes were

collected at day 30 after tumor implantation from ID8 bearing mice with or without TAMs administration. **b** Representative pictures of peritoneal lavages obtained from ID8 bearing mice (*n* = 3 mice per group). **c** Spheroids from peritoneal washes were harvested and evaluated by H&E staining (*n* = 3 mice per group and an average of 5 fields acquired from each sample). Scale bars: 100μm. **d**-**e** Quantification of $Ki67^+$ and $CD68^+$ cells in spheroids (*n* = 5 mice per group and 10 confocal images acquired from each sample). **f** Representative FACS images of infiltrated TAMs in peritoneal cavity. **g** Proportion of $CD11b^+F4/80^+$ macrophages on $CD45^+$ infiltrating cells were quantified (*n* = 5 mice per group). **h** Schematics for LC treatment. **i** Representative FACS images of infiltrated $CD11b^+F4/80^+$ macrophages in peritoneal cavity. **j**-**k** Quantification of $Ki67^+$ cells and $CD68^+$ cells (in spheroid populations) (*n* = 3 mice per group and 10 confocal images acquired from each sample). **l**-**m** Quantification of total number and size of spheroids retrieved from peritoneal cavity (*n* = 5 mice per group and an average of 10 fields acquired from each sample). **n** SRB assay showing proliferation rates of ID8 cells in response to 10μg/ml of LC at different time points (*n* =3 biologically independent samples per group). **o** Quantification of infiltrated $CD3^+$ T cells in peritoneal cavity (*n* =3 mice per group). All data are representative of at least two independent experiments with similar results. Data are presented as mean ± SEM, unpaired two-sided Student's *t* test with no correction for multiple comparison,*$P < 0.05$, **$P < 0.01$, ***$P < 0.001$. Source data are provided as a Source Data file.

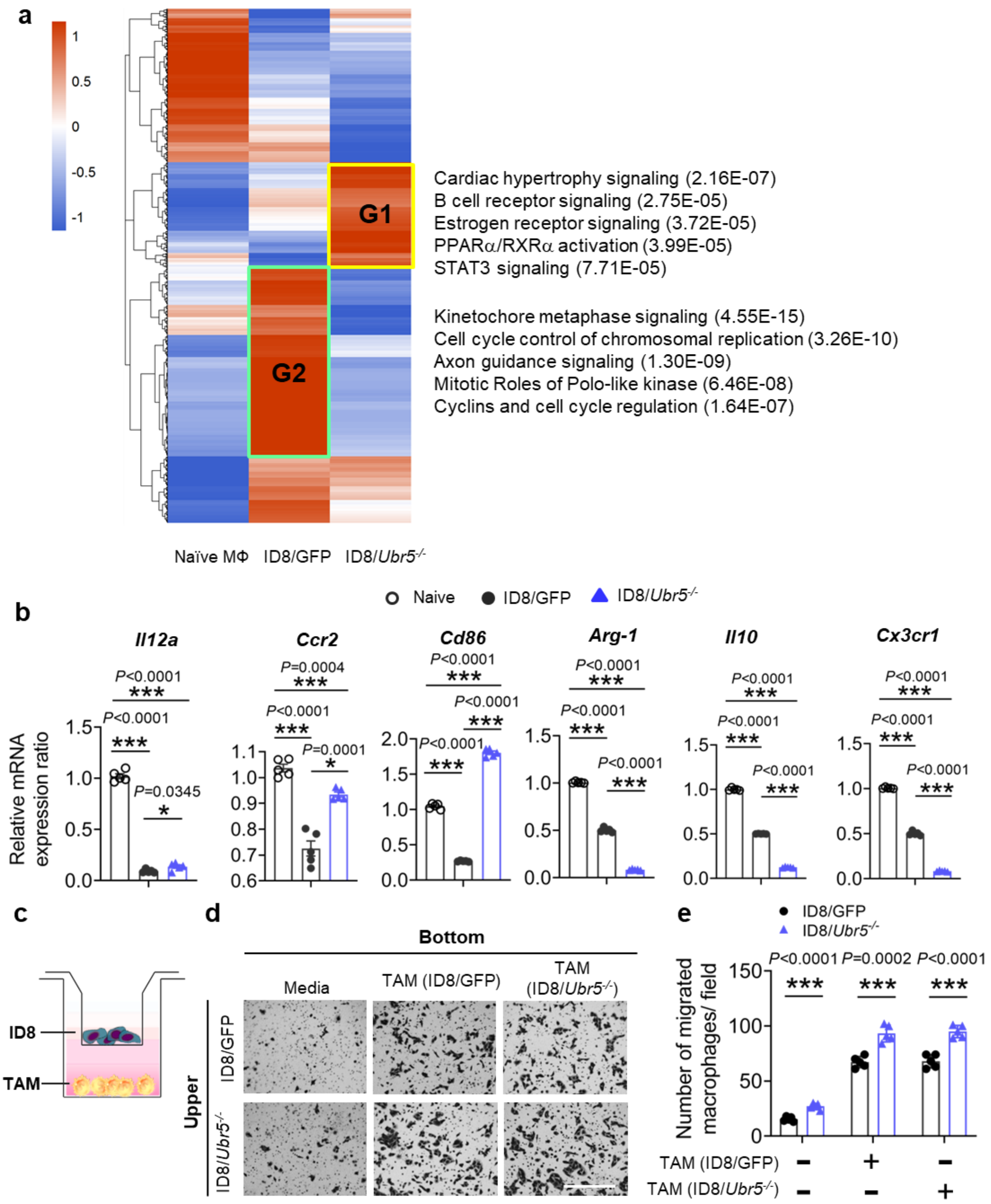

Supplementary Fig.6: UBR5-regulated, paracrine TAM immunosuppressive activities. Figure supplementary to main Figure 4

a RNA-seq analysis of peritoneal macrophages from non-tumor-bearing mice (naïve), ID8/GFP tumor-bearing mice and *Ubr5*$^{-/-}$ tumor bearing mice. All genes with statistically significant expression changes (log2 Fold Change>1) are included in this “heatmap”, highlighting two groups of genes (G1 and G2) that were activated exclusively in *Ubr5*$^{-/-}$ tumor or WT tumor carrying mouse-derived TAMs, respectively. The activated pathways in G1 and G2 based on Ingenuity Pathway Analysis (IPA) are illustrated. Numbers in the brackets indicate the degree of statistical significance. **b**

TAMs were isolated from tumor bearing mice on 5 weeks after tumor implantation and subjected to qRT-PCR. The mRNA levels of M1/M2 subtype markers were evaluated (*n* =5 biologically independent samples per group). **c**-**e** Isolated TAMs from ID8 tumor bearing mice were seeded at the bottom and 2×10^4 starved ID8 cells were loaded into Transwell chamber for 24hrs. **c** Schematics of migration Transwell assay. **d-e** Representative micrographs and quantitative values of migrated ID8 cells (*n* =5 biologically independent samples per group) Scale bars: 200μm. Data in **b**-**e** are representative of three independent experiments with similar results. Data are presented as mean ± SEM, unpaired two-sided Student's *t* test with no correction for multiple comparison, ***$P < 0.001$. Source data are provided as a Source Data file.

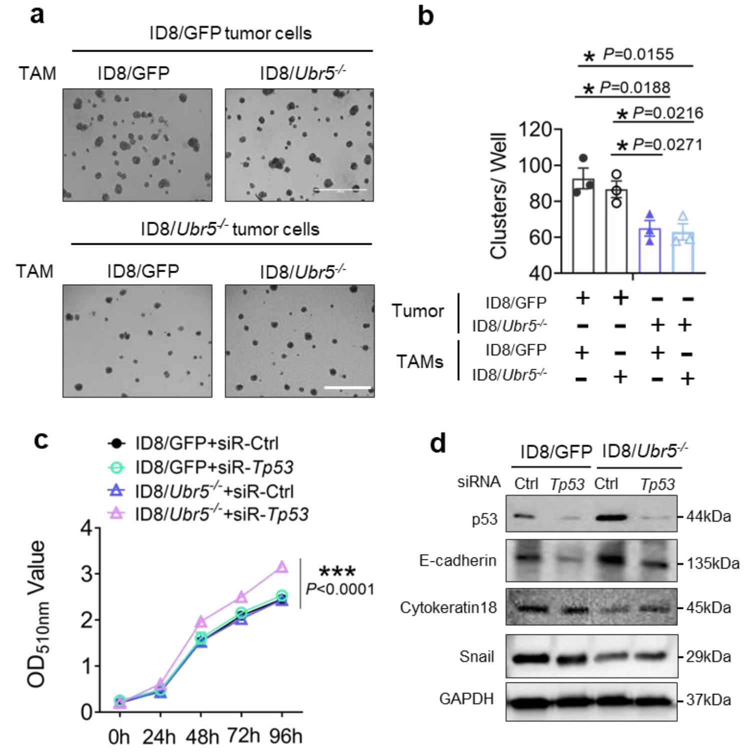

Supplementary Fig.7: UBR5-regulated spheroid formation and p53 expression in ID8. Figure supplementary to main Figure 5

a-c TAMs isolated from ID8 bearing mice were co-cultured with ID8 in a 3D coculture system. **a** Representative images of spheroids. Scale bars: 400μm. **b** The number of spheroids was statistically evaluated ($n = 3$ biologically independent samples per group). **c** nockingdown *Tp53* in ID8/*Ubr5*$^{-/-}$ promoted cell proliferation. Sulforhodamine B (SRB) assay showing growth rates of ID8/*Ubr5*$^{-/-}$ after p53 silencing ($n = 3$ biologically independent samples per group). **d** Western blotting showing the expression of E-cadherin, cytokeratin 18, and Snail after *Tp53* knocking down in ID8 cells. All data are representative of three independent experiments with similar results. Data are presented as mean ± SEM, unpaired two-sided Student's *t* test with no correction for multiple comparison,*$P < 0.05$, ***$P < 0.001$. Source data are provided as a Source Data file.

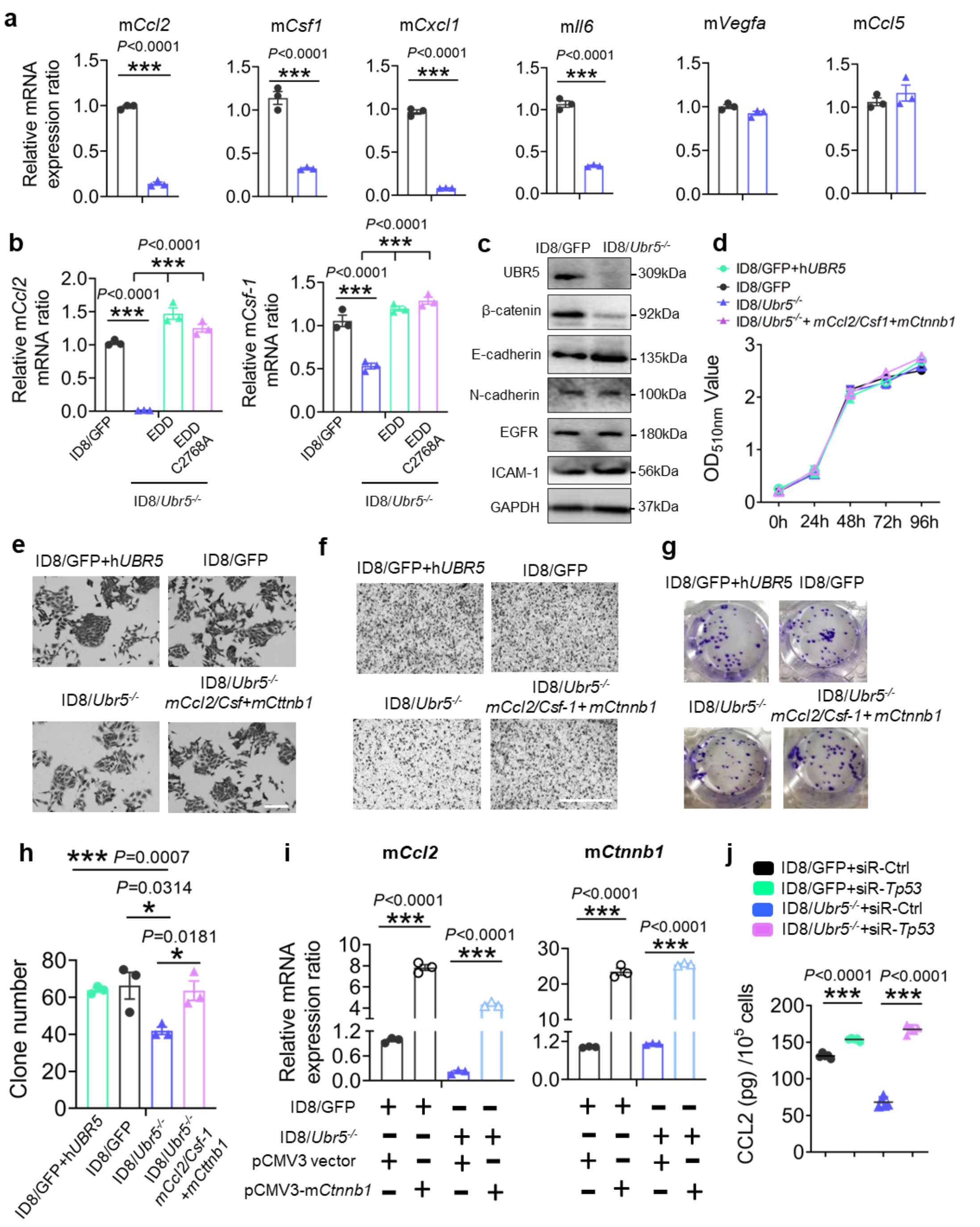

Supplementary Fig. 8: *Ubr5* deficiency reduces paracrine crosstalk between OC and TAMs, and decreases cell-autonomous β-catenin signaling. Figure supplementary to main Figure 6

a The mRNA levels of genes involved in OC-TAMs crosstalk (*Ccl2*, *Csf1*, *Cxcl1*, *Il6*, *Vegfa*, and *Ccl5*) in ID8 cells were evaluated by qRT-PCR ($n = 3$ biologically independent samples per group). **b** mRNA expression of *mCcl2* and *mCsf-1* in ID8/GFP, ID8/*Ubr5*$^{-/-}$, EDD or EDD-C2768A reconstituted ID8/*Ubr5*$^{-/-}$ cells was

evaluated by qRT-PCR (*n* = 3 biologically independent samples per group). **c** Protein expression of β-catenin, E-cadherin, N-cadherin, and EGRF were assessed by Western Blot. **d** Sulforhodamine B (SRB) assay showing growth rates (*n* = 3 biologically independent samples per group). **e** Representative micrographs of cell morphology. Scale bars: 200μm. **f** Transwell migration assay of peritoneal macrophages in the presence of ID8 cells. Scale bars: 400μm. **g**-**h** Representative images and quantified values of clonogenic assay with 100 cells/well (*n* = 3 biologically independent samples per group). **i** Transient transfection of *Ctnnb1* in ID8 cells induced *Ccl2* mRNA expression in ID8 cells (*n* = 3 biologically independent samples per group). **j** Knocking down *Tp53* in ID8 cells increased CCL2 production (*n* = 5 biologically independent samples per group). All data are representative of at least two independent experiments with similar results. Data are presented as mean ± SEM, unpaired two-sided Student's *t* test with no correction for multiple comparison,*$P < 0.05$, ***$P < 0.001$. Source data are provided as a Source Data file.

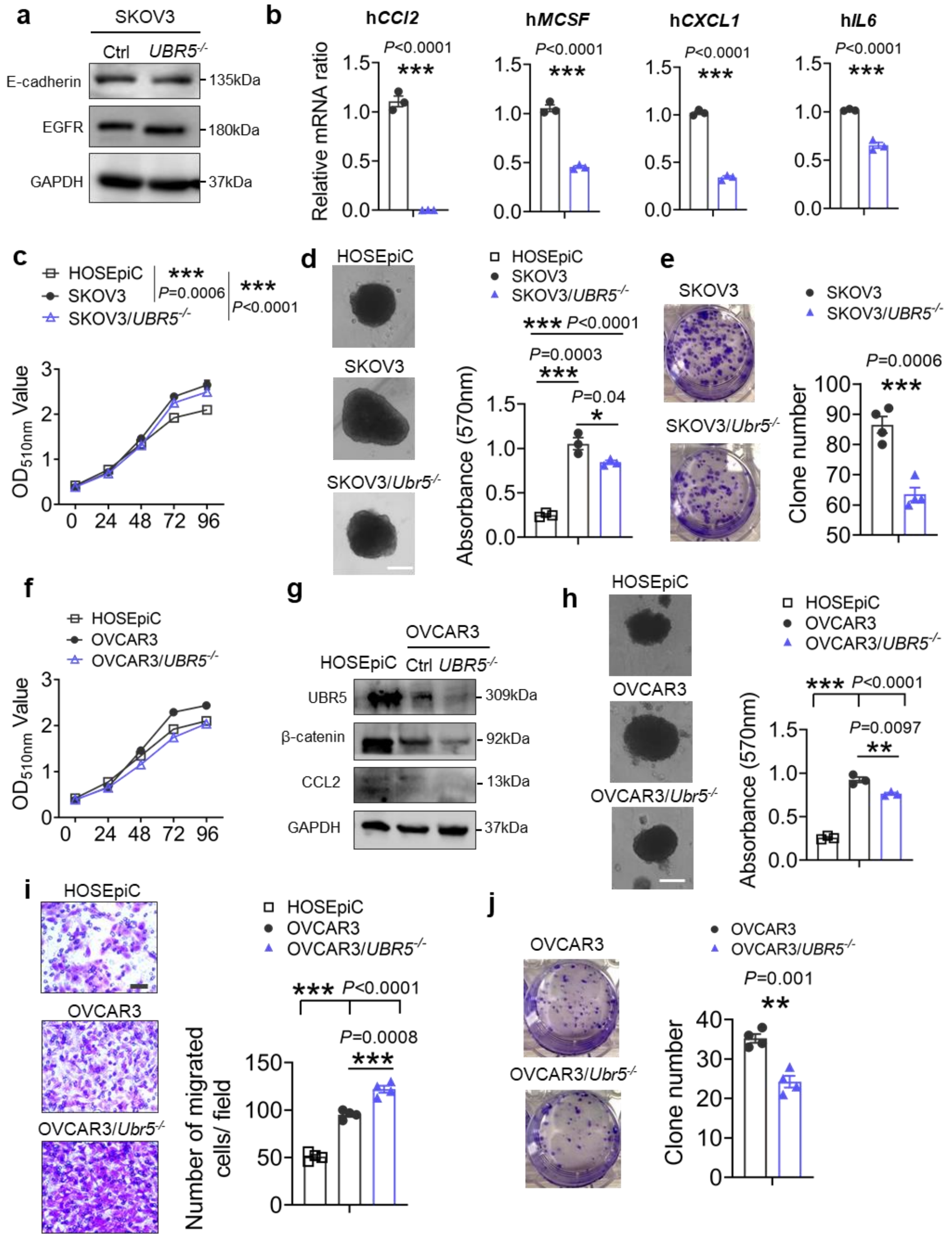

Supplementary Fig.9: *UBR5* depletion leads to reduced macrophage infiltration in human ovarian cancer. Figure supplementary to main Figure 7

a Protein expression of E-cadherin and EGFR were evaluated by western blot. **b** Reduced expression of *CCL2*, *MCSF*, *CXCL1*, *IL6* in SKOV3/*UBR5*$^{-/-}$ were determined at mRNA level by qRT-PCR ($n = 3$ biologically independent samples per group). **c** Sulforhodamine B (SRB) assay showing growth rates of HOSEpiC vs SKOV3 cells ($n = 3$ biologically independent samples per group). **d** Representative images and quantification of SKOV3 spheroid formation ($n = 3$ biologically

independent samples per group). Scale bars: 200μm. **e** Representative micrographs and quantitation of clonogenic assay for SKOV3 ($n = 4$ biologically independent samples per group). **f** Sulforhodamine B (SRB) assay showing growth rates of HOSEpiC vs OVCAR3 cells ($n = 3$ biologically independent samples per group). **g** Deletion of *UBR5* in OVCAR3 cells was verified by western blot. Reduced β-catenin in OVCAR3/*UBR5*$^{-/-}$ was determined at protein level, human ovarian surface epithelial cells HOSEpiC were used as control. **h** Representative images and quantification of OVCAR3 spheroid formation ($n = 3$ biologically independent samples per group). Scale bars: 200μm. **i** Representative micrographs and quantitation of Transwell -migration assay for OVCAR3.Scale bars: 50μm ($n = 4$ biologically independent samples per group and an average of 5 fields acquired from each sample). **j** Representative micrographs and quantitation of clonogenic assay for OVCAR3 ($n = 4$ biologically independent samples per group). All data are representative of at least two independent experiments with similar results. Data are presented as mean ± SEM, unpaired two-sided Student's *t* test with no correction for multiple comparison, $^{**}P < 0.01$, $^{***}P < 0.001$. Source data are provided as a Source Data file.

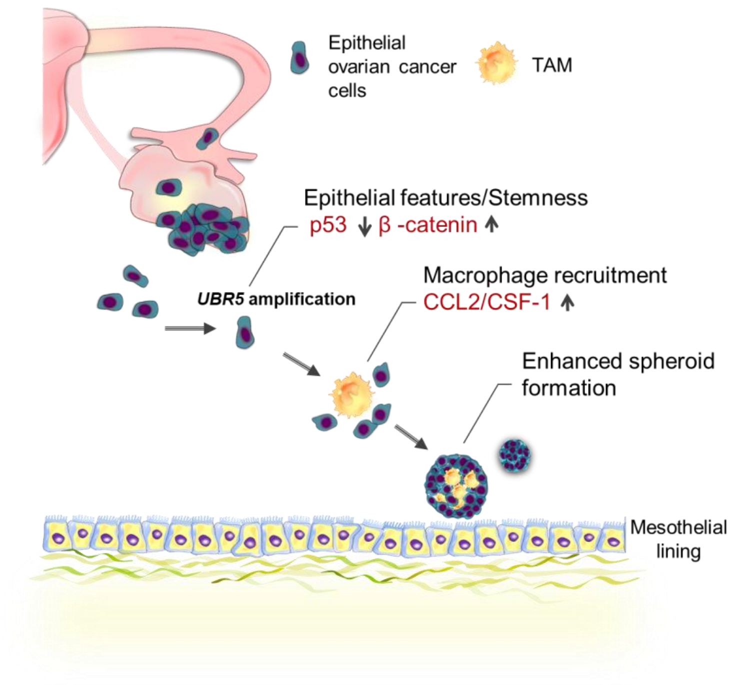

Supplementary Fig.10: Schematic illustration of how tumor-derived UBR5 promotes ovarian cancer progression. Figure supplementary to Discussion

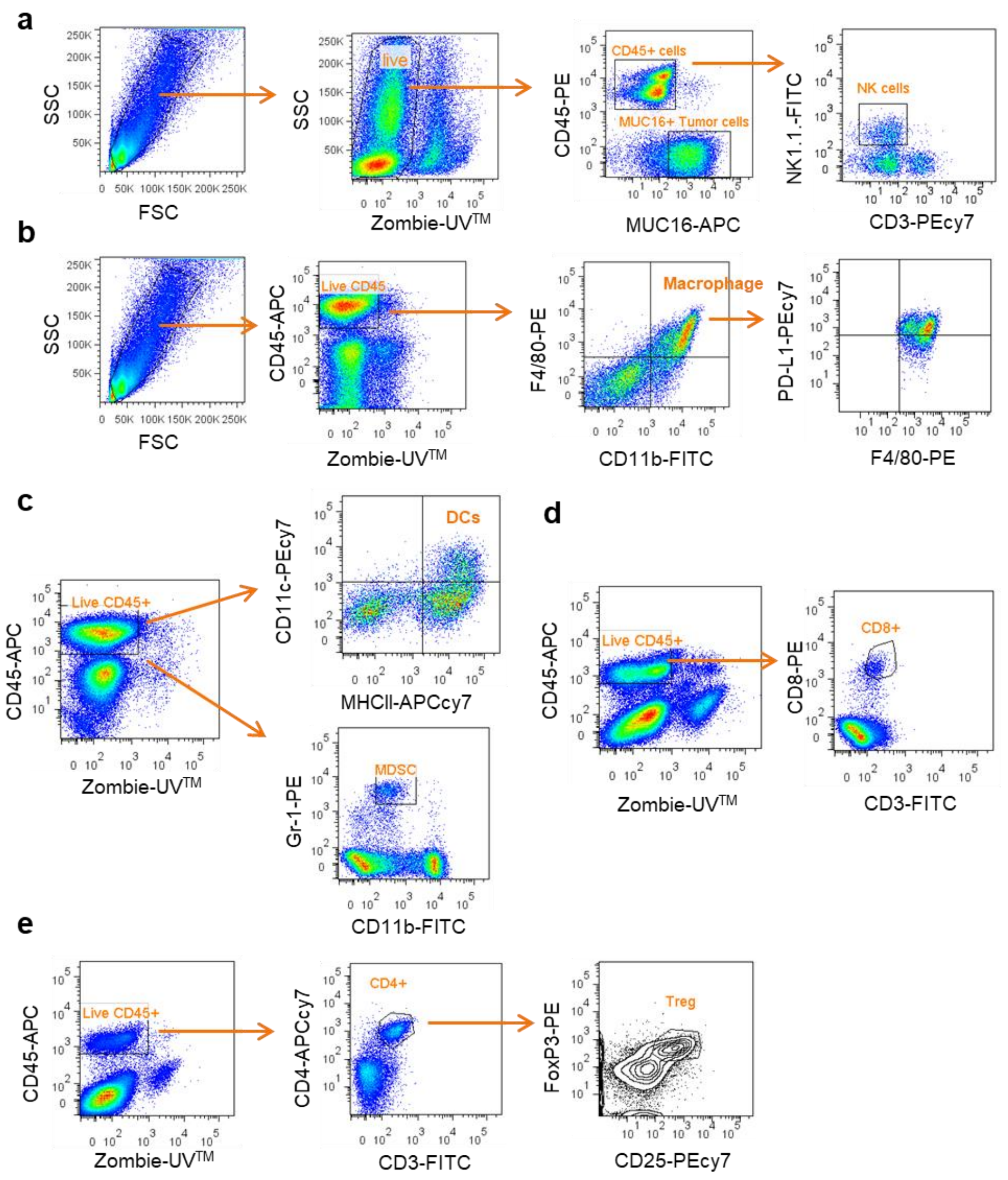

Supplementary Fig.11: FACS Gating strategy for tumor cells and immune subsets from peritoneum.

Following gating to live cells by Zombie-UV fixable viability stain, ID8 tumor cells were gated for $CD45^{-}MUC16^{+}$ cells presented on Fig.1i, 2d,3a, f, Supplementary Fig.2g, 3f, 4g,h, 5a; infiltrating immune cells were gated for $CD45^{+}$ cells. NK cells were identified as $CD45^{+}NK1^{+}CD3^{-}$ presented on Supplementary Fig.4e, f (**a**). Macrophages were identified as $CD45^{+}CD11b^{+}F4/80^{+}$ presented on Fig2e, f, 3e, 4c, d, 7i, Supplementary Fig.5f, g, and I (**b**); DCs and MDSCs were identified as $CD45^{+}MHCII^{+}CD11c^{+}$, $CD45^{+}CD11b^{+}Gr\text{-}1^{+}$, respectively presented in Supplementary Fig.4i (**c**).CD8+ T cells were gated as $CD45^{+}CD3^{+}CD8^{+}$ presented in Fig.2b, Supplementary Fig.4b (**d**). CD4+ T cells were gated as $CD45^{+}CD3^{+}CD4^{+}$ and Tregs were gated on $CD4^{+}$ T cells as $Foxp3^{+}CD25^{+}$ ($CD4^{+}$T) cells presented in Fig2a, c, Supplementary Fig.4a, c (**e**).

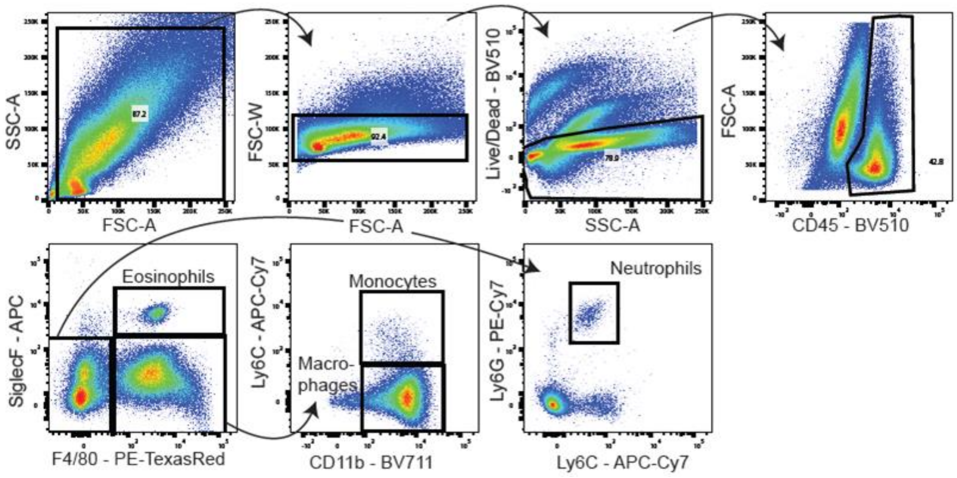

Supplementary Fig.12: FACS Gating strategy for counting the absolute number of myeloid cell population from peritoneum of ID8 tumor bearing mice.

Following gating to include singlets (FSC-H vs. FSC-A) and live cells by Live/Dead-BV510, tumor infiltrating myeloid cells were gated for CD45^{+}cells. Macrophages were identified as F4/80^{+}CD11b^{+}Siglec F^{-}Ly6C^{-}; Monocytes were identified as F4/80^{+}CD11b^{+} Ly6C^{+} Siglec F^{-}; Eosinophils were identified as Siglec F^{+} F4/80^{+}, Neutrophils were identified as F4/80^{-} Siglec F^{-}Ly6G^{+}Ly6C^{+} presented in Supplementary Fig4i.

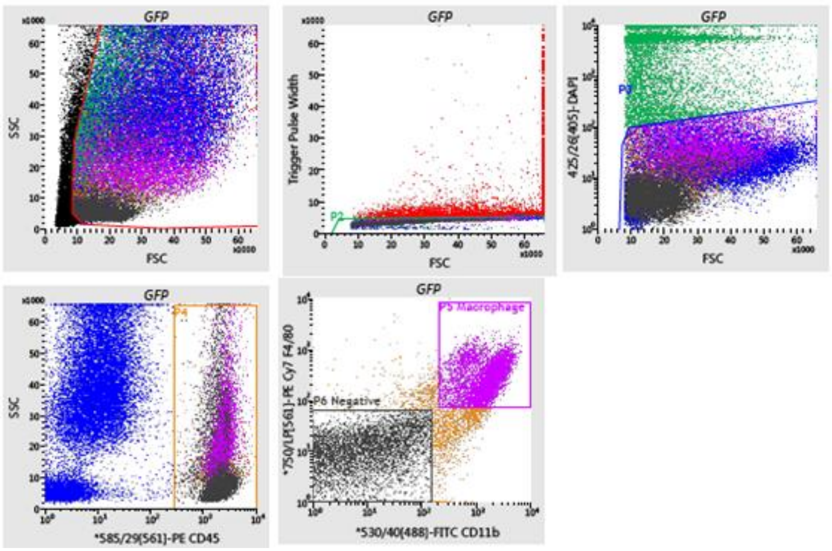

Supplementary Fig.13: Gating strategy for tumor associated macrophages (TAMs) sorting from peritoneum of ID8 tumor bearing mice. Figure supplementary to Figure 3,4g and 7k.

Following gating to include singlets (FSC vs. SSC) and live cells by DAPI, tumor associated macrophages were identified as F4/80^{+}CD11b^{+} cells and sorted with Becton-Dickinson Influx.

Supplementary Table 1: Complete blood count (CBC) and liver function parameters in tumor bearing mice with intrabursal injection of ID8. Table supplementary to main Figure 1

**Complete blood count**	ID8/GFP	ID8/*Ubr5*$^{-/-}$	*p* value	Reference range
RBC (M/uL)	10.32±0.12	10.45±0.16	n.s.	7.14-12.20
HGB (g/dL)	15.23±0.06	15.43±0.15	n.s.	10.8-19.2
HCT (%)	50.06±0.81	49.47±1.50	n.s.	37.2-62.0
MCV (fL)	48.53±0.25	47.77±0.59	n.s.	42.6-56.0
MCH (pg)	14.77±0.15	14.70±0.10	n.s.	11.7-16.8
MCHC (g/dL)	30.43±0.50	30.80±0.20	n.s.	24.6-35.9
RDW-SD (fL)	28.30±0.50	27.37±0.78	n.s.	----
RET (%)	4.13±0.09	3.34±0.18	**(0.0024)	2.56-4.56
PLT (K/uL)	836.00±204.73	869.00±147.31	n.s.	565-2159
PDW (fL)	7.43±0.40	7.27±0.32	n.s.	----
MPV (fL)	6.17±0.38	5.97±0.15	n.s.	4.3-6.1
WBC# (K/uL)	4.90±1.32	5.75±0.53	n.s.	3.90-13.96
NEUT (%)	11.80±2.82	8.40±0.79	n.s.	7.36-28.59
LYMPH (%)	85.93±3.36	87.10±3.58	n.s.	61.26-87.12
MONO (%)	0.50±0.00	0.37±0.12	n.s.	2.18-11.02
EO (%)	1.77±0.61	1.93±0.57	n.s.	0.13-4.51
**Liver chemistry**				
ALP (U/L)	106.33±17.24	105.67±8.50	n.s.	105-370
ALT (U/L)	32.67±15.04	30.67±7.37	n.s.	27-195
AST (U/L)	55.67±14.29	50.67±19.55	n.s.	54-77
LDH (mg/dL)	364.33±115.31	285.67±143.17	n.s.	----
TBIL (mg/dL)	0.17±0.06	0.17±0.06	n.s.	0.2-0.6
IBIL (mg/dL)	0.17±0.06	0.17±0.06	n.s.	----
TP (g/dL)	5.03±0.21	4.800±0.00	n.s.	4.8-7.2
ALB (g/dL)	3.20±0.10	3.00±0.00	*(0.0257)	2.4-4.3
GLOB (g/dL)	1.83±0.12	1.70±0.00	n.s.	1.7-2.2
A/G Ratio	1.73±0.06	1.80±0.00	n.s.	----
CHOL (mg/dL)	73.33±2.31	51.33±4.93	**(0.0022)	55-169
TRIG (mg/dL)	123.33±22.01	90.67±15.82	n.s.	67-289

7 weeks after tumor implantation, blood samples were collected for complete blood count (CBC) and liver function test. RBC: red blood cell; HGB: hemoglobin; HCT: hematocrit; MCV: mean corpuscular volume; MCH: mean corpuscular hemoglobin; MCHC: mean corpuscular hemoglobin concentration; RDW: red blood cell distribution width; RET: reticulocyte; PLT: platelet count; PDW: platelet distribution width; MPV: mean platelet volume; WBC: white blood cell; NEUT: neutrophil; LYMPH: lymphocyte; MONO: monocyte; EO: eosinophil. ALP: alakline phosphatase; ALT: alanine transaminase; AST: aspartate transaminase; LDH: lactate dehydrogenase; TBIL: total bilirubin; IBIL:indirect bilirubin; TP: total protein; ALB: albumin; GLOB: globulin-calculated; CHOL: cholesterol; TRIG: triglycerides. Data are presented as mean±SD. Statistical significance was assessed based on unpaired two-tailed Student's t-test, $*P < 0.05$, $**P < 0.01$.

Supplementary Table 2: Complete blood count (CBC) and liver function parameters in tumor bearing mice with intraperitoneal (i.p.) injection with or w/o cisplatin treatment. Table supplementary to main Figure 8

	**PBS**						**Cisplatin**					
	Average±SD			*p* value			Average±SD			*p* value		
**Complete blood count**	ID8/GFP(#1)	ID8/*Ubr5*$^{-/-}$ (#2)	ID8/GFP +h*UBR5*(#3)	#1vs #2	#1vs#3	#2vs #3	ID8/GFP(#1)	ID8/*Ubr5*$^{-/-}$ (#2)	ID8/GFP +h*UBR5*(#3)	#1vs #2	#1vs #3	#2 vs #3
RBC (M/uL)	9.23±0.38	9.98±0.74	5.09±1.40	n.s.	*** (0.0004)	*** (<0.0001)	9.71±0.38	8.92±0.50	8.52±1.02	n.s.	n.s.	n.s.
Hemoglobin (g/dL)	15.00±1.4	14.50±0.96	6.90±1.50	n.s.	** (0.0022)	** (0.0016)	13.53±1.51	12.00±1.49	13.30±1.72	n.s.	n.s.	n.s.
Hematocrit (%)	52.00±3.64	51.43±1.70	25.8±4.86	n.s.	** (0.0012)	*** (0.0005)	49.13±5.05	43.8±4.56	47.9±3.56	n.s.	n.s.	n.s.
MCV (fL)	51.53±3.23	49.53±1.58	52.87±1.4	n.s.	n.s.	n.s.	49.47±2.99	48.13±3.56	45.27±1.68	n.s.	n.s.	n.s.
MCH (pg)	13.90±0.20	14.27±0.45	13.70±1.04	n.s.	n.s.	n.s.	14.40±0.44	14.03±0.06	14.57±0.49	n.s.	n.s.	n.s.
MCHC (g/dL)	30.56±0.45	30.67±0.32	28.20±2.42	n.s.	n.s.	n.s.	30.97±0.90	30.80±0.53	31.33±0.76	n.s.	n.s.	n.s.
RDW-SD (fL)	27.50±1.25	28.30±0.60	33.03±8.23	n.s.	n.s.	n.s.	29.00±1.31	26.17±1.45	27.50±0.70	n.s.	n.s.	n.s.
RDW-CV (%)	24.63±0.56	24.60±0.44	24.40±2.05	n.s.	n.s.	n.s.	24.33±0.91	24.03±1.30	23.86±0.25	n.s.	n.s.	n.s.
RET (%)	4.11±0.53	4.37±0.61	7.98±0.47	n.s.	*** (0.0007)	** (0.0012)	4.06±0.63	3.98±0.45	4.78±0.68	n.s.	n.s.	n.s.
PLT (K/uL)	849.67± 151.08	883.00± 153.52	727.33± 88.09	n.s.	n.s.	n.s.	875.01± 225.43	766.20± 125.46	944.07± 227.58	n.s.	n.s.	n.s.
PDW (fL)	6.60±0.44	6.63±0.76	6.70±0.10	n.s.	n.s.	n.s.	6.43±0.15	6.23±0.06	6.56±0.32	n.s.	n.s.	n.s.
MPV (fL)	5.57±0.12	5.67±0.16	6.36±0.83	n.s.	n.s.	n.s.	5.60±0.17	5.50±0.14	5.83±0.35	n.s.	n.s.	n.s.
WBC# (K/uL)	5.13±0.58	4.99±0.74	5.24±0.70	n.s.	n.s.	n.s.	4.39±0.34	4.31±0.07	5.13±0.94	n.s.	n.s.	n.s.
NEUT (%)	11.4±10.87	8.53±2.08	18.83±7.54	n.s.	n.s.	n.s.	11.60±0.28	11.89±1.00	10.40±0.72	n.s.	n.s.	n.s.
LYMPH (%)	88.60±3.41	88.57±3.13	80.00±7.54	n.s.	n.s.	n.s.	85.80±0.50	75.97±5.82	85.70±0.40	**(0.0065)	n.s.	**(0.0083)
MONO (%)	0.70±0.10	0.67±0.15	0.60±0.20	n.s.	n.s.	n.s.	0.50±0.07	0.53±0.15	0.70±0.10	n.s.	n.s.	n.s.
EO (%)	2.00±0.10	1.80±0.10	1.47±0.45	n.s.	n.s.	n.s.	1.97±0.42	2.00±0.32	1.40±0.55	n.s.	n.s.	n.s.
**Liver chemistry**												
ALP (U/L)	116.33±5.51	106.33±4.51	73.67±9.61	n.s.	**(0.0026)	**(0.0060)	133±11.27	96.33±13.01	120.67±9.50	*(0.021)	n.s.	**(0.0094)
ALT (U/L)	26.00±4.00	24.00±6.00	33.67±15.37	n.s.	n.s.	n.s.	26.33±3.21	38.33±3.21	29.67±9.01	*(0.0102)	n.s.	n.s.
AST (U/L)	50.33±5.50	52.00±10.44	50.67±6.43	n.s.	n.s.	n.s.	49.67±4.51	60.33±6.21	52.00±10.44	n.s.	n.s.	n.s.
LDH (mg/dL)	347.33± 35.57	336.33± 20.55	305.67± 58.29	n.s.	n.s.	n.s.	341.00± 19.16	353.00± 116.66	366.33± 43.29	n.s.	n.s.	n.s.

TBIL (mg/dL)	0.13±0.06	0.23±0.06	0.2±0.1	n.s.	n.s.	n.s.	0.2±0.1	0.2±0.00	0.13±0.06	n.s.	n.s.	n.s.
IBIL (mg/dL)	0.13±0.06	0.13±0.06	0.1±0.00	n.s.	n.s.	n.s.	0.13±0.06	0.2±0.00	0.2±0.1	n.s.	n.s.	n.s.
TP (g/dL)	5.10±0.10	5.01±0.06	4.87±0.31	n.s.	n.s.	n.s.	4.97±0.15	5.17±0.06	4.93±0.15	n.s.	n.s.	n.s.
ALB (g/dL)	3.20±0.10	3.30±0.10	2.70±0.10	n.s.	**(0.0036)	**(0.0018)	3.23±0.06	3.23±0.06	3.23±0.12	n.s.	n.s.	n.s.
GLOB (g/dL)	1.77±0.12	1.87±0.06	1.77±0.06	n.s.	n.s.	n.s.	1.80±0.10	1.93±0.06	1.83±0.12	n.s.	n.s.	n.s.
A/G Ratio	1.77±0.12	1.87±0.06	1.70±0.17	n.s.	n.s.	n.s.	1.83±0.12	1.67±0.06	1.77±0.06	n.s.	n.s.	n.s.
CHOL (mg/dL)	70.00±8.54	66.33±8.39	86.67±25.00	n.s.	n.s.	n.s.	72.33±4.11	74.67±10.97	76.00±12.77	n.s.	n.s.	n.s.
TRIG (mg/dL)	78.00±8.72	84.00±9.16	75.67±12.34	n.s.	n.s.	n.s.	82.67±15.01	87.33±9.71	77.67±22.14	n.s.	n.s.	n.s.

Blood samples were collected 5 weeks after tumor inoculation (1 week following discontinuation of cisplatin treatment) for CBC and LFT test. Data are presented as mean±SD. Statistical significance was assessed based on unpaired two-tailed Student's t-test, **P* < 0.05, ***P* < 0.01, ****P* < 0.001

Supplementary Table 3: Demographic characteristics of patients with epithelial ovarian cancer. Table supplementary to main Figure 7

**Characteristic**	**No. of patients (n=50)**	**Expression of UBR5 （IRS）**			***P***
Age at diagnosis (years)		0-3	4--6	8--12	
Mean	53				
Media	52.5				
Range	28-77				
**FIGO stage**					0.449
ⅠA or ⅠC	9 (18%)	4	2	3	
Ⅱ	5 (10%)	3	1	1	
ⅢA or ⅢB	3 (6%)	2	1	0	
ⅢC	17 (34%)	8	7	2	
Ⅳ	10 (20%)	8	2	0	
Unknown	6 (12%)	5	0	1	
**Histology Type**					0.323
Seruous adenocarcinoma	29 (58%)	16	8	5	
Mucoid adenocarcinoma	8 (16%)	6	0	2	
Clear cell adenocarcinoma	10 (20%)	5	1	4	
Endometrioid adenocarcinoma	2 (4%)	2	0	0	
**Primary tumor size (cm)**					0.163
<5	16	12	3	1	
≥5	34	16	11	7	
**Debulking status**					0.257
No residual	27	15	5	7	
Regional recurrence	13	5	7	1	
Never disease free	6	4	1	1	
Unknown	4	2	2	0	

The differences in demographic characteristics of OC patients were analyzed by the $\chi 2$ test.

Supplementary Table 4: Antibody list.

Antibody name	Company	Host Species	Cat No.	Usage	Dilution
UBR5/EDD	Novus bio	Rabbit	NB100-1591	WB	1:500
EDD	Santa Cruz	Goat	sc-9562	IHC	1:500
GAPDH	Santa Cruz	Rabbit	sc-25778	WB	1:1000
β-Catenin	Cell signaling	Rabbit	9562	WB	1:500
Cytokeratin 18	Santa Cruz	Mouse	sc-51582	WB	1:500
β-Catenin	Santa Cruz	Mouse	sc-7963	IF	1:500
CA125 (MUC16)	Invitrogen	Rabbit	MA5-32321	IF	1:200
Vimentin	Santa Cruz	Mouse	sc-6260	IF/WB	1:200
ZEB1	Novus bio	Rabbit	NBP1-05987	WB	1:500
ZEB2	Santa Cruz	Rabbit	sc-48789	WB	1:500
PARP	Cell signaling	Rabbit	9542S	WB	1:400
P53	R&D	Mouse	MAB1355	WB	1:200
P53	Santa Cruz	Mouse	sc-126	WB	1:400
Snail	Cell signaling	Rabbit	3879	WB	1:500
HDAC1	Santa Cruz	Mouse	sc-81598	WB	1:500
E-cadherin	Novus bio	Mouse	NBP2-19051	WB	1:400
N-cadherin	Cell signaling	Rabbit	4061	WB	1:200
EGF Receptor	Cell signaling	Rabbit	2232	WB	1:500
ICAM-1	Santa Cruz	Mouse	sc-107	WB	1:500
CCL2/MCP-1	Invitrogen	Mouse	MA5-17040	WB	1:200
Ki67	Vector Laboratories	Rabbit	VP-RM04	IF	1:100
CD68	Santa Cruz	Mouse	sc-20060	IF/IHC	1:100
CD3-FITC	Biolegend	Rat	100204	FACS	1:200
CD3-PE/Cyanine7	Biolegend	Rat	100220	FACS	1:200
CD45-APC/Cyanine7	Biolegend	Rat	103116	FACS	1:200
CD45-BV510	Biolegend	Rat	103138	FACS	1:200
CD8a-PE	Biolegend	Rat	100708	FACS	1:200
CD4-APC/Cyanine7	Biolegend	Rat	100414	FACS	1:200
CD11b-FITC	Biolegend	Rat	101206	FACS	1:200
CD11b-BV711	Biolegend	Rat	101242	FACS	1:200
NK1.1-FITC	Biolegend	Mouse	108706	FACS	1:200
F4/80-PE	Biolegend	Rat	123110	FACS	1:200
Ly6G-PE/Cyanine7	Biolegend	Rat	127618	FACS	1:200
Ly6C-APC/Cyanine7	Biolegend	Rat	128026	FACS	1:200
CD11c-PE/Cyanine7	Biolegend	Rat	117318	FACS	1:200
I-A/I-E (MHCll) -APC/Cyanine7	Biolegend	Rat	107628	FACS	1:200
CD274(PD-L1)-PE/Cyanine7	Biolegend	Rat	124313	FACS	1:200
Siglec F(CD170)-APC	Biolegend	Rat	155508	FACS	1:200
CD45-PE	eBioscience	Rat	2-0451-82	FACS	1:200

CD4-PE/Cyanine7	eBioscience	Rat	25-0041-82	FACS	1:200
FOXP3-PE	eBioscience	Rat	12-5773-82	FACS	1:100
CD25-PE/Cyanine7	eBioscience	Rat	25-0251-82	FACS	1:200
Arginase-1-PE	eBioscience	Rat	12-3697-82	FACS	1:100

Supplementary Table 5: **RT-PCR primer list**.

Gene name	Species	Forward Sequence	Reverse Sequence
Ubr5	Mouse	CAATCACGCCGCGTTTCTT	GTCCTTCCTGGCCTAGAGGT
Gapdh	Mouse	GTCCTTCCTGGCCTAGAGGT	GATGCAGGGATGATGTTCTG
Ccl2	Mouse	TTAAAAACCTGGATCGGAACCAA	GCATTAGCTTCAGATTTACGGGT
Ccr2	Mouse	ATCCACGGCATACTATCAACATC	CAAGGCTCACCATCATCGTAG
Tp53	Mouse	TGGAAGACTCCAGTGGGAAC	CTTCTTCTGTACGGCGGTCT
Csf-1	Mouse	GGAGTATCACCGAGGAGGTG	ATTTGGCACGAGGTCTCCAT
Arg1	Mouse	GGACCTGGCCTTTGTTGATG	AACTGCCAGACTGTGGTCTC
Vegfa	Mouse	CTGCTGTACCTCCACCATGC	TCACTTCATGGGACTTCTGCTCT
Il10	Mouse	AAGGACCAGCTGGACAACAT	TTCACAACCCTTGACTGCTG
Cx3cr1	Mouse	GACCGGTGTCACCATTAG	AGTCACCCAGACACTCGTTG
Ccl5	Mouse	TGCCCACGTCAAGGAGTATT	TTCTTCTCTGGGTTGGCACA
Cxcl1	Mouse	ACCCAAACCGAAGTCATAGC	GTGCAATCAGAGCAGTCTGT
Il12a	Mouse	AAATGAAGCTCTGCATCCTGC	TCACCCTGTTGATGGTCACG
Il6	Mouse	CCCAATTTCCAATGCTCTCC	TGAATTGGATGGTCTTGGTCC
UBR5	Human	CACCTTAGGAAGCCGCTGGA	CGATCCCGTCCTCTCCGAAT
CCL2	Human	AAGATCTCAGTGCAGAGGCT	TGAACCCACTTCTGCTTGGG
MCSF	Human	TCGGAGGCCTCTTGTTCTAC	TCCACCTGTCTGTCATCCTG
CXCL1	Human	GCCAGTGCTTGCAGACCCT	GGCTATGACTTCGGTTTGGG
IL6	Human	AATTCGGTACATCCTCGACGG	GGTTGTTTTCTGCCAGTGCC
GAPDH	Human	GAAGGTGGAGGTCGGAGTCA	TGGAATCATATTGGAACATGT
pac	yeast	CGCAGCAACAGATGGAAGG	GGAGGTCTCCAGGAAGGC

Supplementary References

1 O'Brien, P. M. *et al.* The E3 ubiquitin ligase EDD is an adverse prognostic factor for serous epithelial ovarian cancer and modulates cisplatin resistance in vitro. *British journal of cancer* **98**, 1085-1093, doi:10.1038/sj.bjc.6604281 (2008).
