# Supplementary material for: Tumor derived UBR5 promotes ovarian cancer growth and metastasis through inducing immunosuppressive macrophages
Source: Nat Commun. 2020 Dec 8;11:6298. doi: 10.1038/s41467-020-20140-0 (PMC7722725; doi:10.1038/s41467-020-20140-0)
Supplement: Supplementary file 3 — Description of Additional Supplementary Files [file 41467_2020_20140_MOESM3_ESM.docx]

**Supplementary data legends**

File Name: Supplementary Data 1

Description: RNA-seq analysis of peritoneal macrophages (CD45^+^CD11b^+^F4/80^+^) retrieved from non-tumor bearing mice (naïve), ID8/GFP tumor bearing mice, and ID8/*Ubr5*^-/-^ tumor bearing mice. Selected genes (log2 fold-change > 1 and adjusted *P*-value < 0.05) defined as G1 in Supplementary Fig.6a that were activated exclusively in macrophages from ID8/*Ubr5^-/-^* tumor bearing mice are listed.

File Name: Supplementary Data 2

Description: RNA-seq analysis of peritoneal macrophages (CD45^+^CD11b^+^F4/80^+^) retrieved from non-tumor bearing mice (naïve), ID8/GFP tumor bearing mice, and ID8/*Ubr5^-/-^* tumor bearing mice. Selected genes (log2 fold-change > 1 and adjusted *P*-value < 0.05) defined as G2 in Supplementary Fig.6a that were activated exclusively in macrophages from ID8/GFP tumor bearing mice are listed.
