# Supplementary material for: Tumor derived UBR5 promotes ovarian cancer growth and metastasis through inducing immunosuppressive macrophages
Source: Nat Commun. 2020 Dec 8;11:6298. doi: 10.1038/s41467-020-20140-0 (PMC7722725; doi:10.1038/s41467-020-20140-0)
Supplement: Supplementary file 6 — Reporting Summary [file 41467_2020_20140_MOESM6_ESM.pdf]

nature research

Corresponding author(s): Xiaojing Ma
Last updated by author(s): Oct 2, 2020

Reporting Summary

Nature Research wishes to improve the reproducibility of the work that we publish. This form provides structure for consistency and transparency in reporting. For further information on Nature Research policies, see our Editorial Policies and the Editorial Policy Checklist.

Statistics

For all statistical analyses, confirm that the following items are present in the figure legend, table legend, main text, or Methods section.

n/a	Confirmed	
☐	☒	The exact sample size (*n*) for each experimental group/condition, given as a discrete number and unit of measurement
☐	☒	A statement on whether measurements were taken from distinct samples or whether the same sample was measured repeatedly
☐	☒	The statistical test(s) used AND whether they are one- or two-sided<br>*Only common tests should be described solely by name; describe more complex techniques in the Methods section.*
☒	☐	A description of all covariates tested
☐	☒	A description of any assumptions or corrections, such as tests of normality and adjustment for multiple comparisons
☐	☒	A full description of the statistical parameters including central tendency (e.g. means) or other basic estimates (e.g. regression coefficient) AND variation (e.g. standard deviation) or associated estimates of uncertainty (e.g. confidence intervals)
☐	☒	For null hypothesis testing, the test statistic (e.g. *F*, *t*, *r*) with confidence intervals, effect sizes, degrees of freedom and *P* value noted<br>*Give P values as exact values whenever suitable.*
☒	☐	For Bayesian analysis, information on the choice of priors and Markov chain Monte Carlo settings
☒	☐	For hierarchical and complex designs, identification of the appropriate level for tests and full reporting of outcomes
☐	☒	Estimates of effect sizes (e.g. Cohen's *d*, Pearson's *r*), indicating how they were calculated

Our web collection on statistics for biologists contains articles on many of the points above.

Software and code

Policy information about availability of computer code

Data collection	Flow cytometry:FACSCalibur (BC Biosciences) or Becton-Dickinson Influx; Confocal microscopy: Zeiss 880 Laser Scanning Confocal Microscope; RT-PCR: QuantStudioTM 6 Flex System (Applied Biosystem); RNA-seq: Illumina Nova Seq6000
Data analysis	Flow cytometry: FlowJo 7.6.1; Microscope: ZEN2 (blue edition)software; ImageJ, RNA-seq gene expression analysis: DEseq2(v1.6.3) , Statistics: GraphPad Prism 8.4.3 software

For manuscripts utilizing custom algorithms or software that are central to the research but not yet described in published literature, software must be made available to editors and reviewers. We strongly encourage code deposition in a community repository (e.g. GitHub). See the Nature Research guidelines for submitting code & software for further information.

Data

Policy information about availability of data

All manuscripts must include a data availability statement. This statement should provide the following information, where applicable:
- Accession codes, unique identifiers, or web links for publicly available datasets
- A list of figures that have associated raw data
- A description of any restrictions on data availability

The RNA seq data generated in this study have been deposited in NCBI GEO database under accession code GSE158911. Figures for the relationship between TP53 mutation and UBR5 gene alterations in serous ovarian carcinoma cohorts (Fig.5j, k) were generated from publicly available datasets from the TCGA (https://www.cbioportal.org/study/summary?id=ov_tcga). Figures The authors declare that all data supporting the findings of this study are available within the article and Supplementary information files and from the corresponding author upon reasonable request. Source data are provided with this paper.

Field-specific reporting

Please select the one below that is the best fit for your research. If you are not sure, read the appropriate sections before making your selection.

☒ Life sciences ☐ Behavioural & social sciences ☐ Ecological, evolutionary & environmental sciences

For a reference copy of the document with all sections, see nature.com/documents/nr-reporting-summary-flat.pdf

Life sciences study design

All studies must disclose on these points even when the disclosure is negative.

Sample size	No sample size calculation was performed. Sample size was determined based on the level and consistency between two different groups. All data sets include at least three biological replicates.
Data exclusions	No data was excluded from the analyses in this study.
Replication	All experiments have been reproduced at least two times, and all attempts at replication were successful with self-consistent results.
Randomization	All allocations were random in this study.
Blinding	The investigators were blinded to group allocation during data collection and analysis.

Reporting for specific materials, systems and methods

We require information from authors about some types of materials, experimental systems and methods used in many studies. Here, indicate whether each material, system or method listed is relevant to your study. If you are not sure if a list item applies to your research, read the appropriate section before selecting a response.

Materials & experimental systems

n/a	Involved in the study	
☐	☒	Antibodies
☐	☒	Eukaryotic cell lines
☒	☐	Palaeontology and archaeology
☐	☒	Animals and other organisms
☐	☒	Human research participants
☒	☐	Clinical data
☒	☐	Dual use research of concern

Methods

n/a	Involved in the study	
☒	☐	ChIP-seq
☐	☒	Flow cytometry
☒	☐	MRI-based neuroimaging

Antibodies

Antibodies used

UBR5/EDD Novus bio Rabbit NB100-1591 WB 1:500
EDD Santa Cruz Goat sc-9562 IHC 1:500
GAPDH Santa Cruz Rabbit sc-25778 WB 1:1000
β-Catenin Cell signaling Rabbit 9562 WB 1:500
Cytokeratin 18 Santa Cruz Mouse sc-51582 WB 1:500
β-Catenin Santa Cruz Mouse sc-7963 IF 1:500
CA125 (MUC16) Invitrogen Rabbit MA5-32321 IF 1:200
Vimentin Santa Cruz Mouse sc-6260 IF/WB 1:200
ZEB1 Novus bio Rabbit NBP1-05987 WB 1:500
ZEB2 Santa Cruz Rabbit sc-48789 WB 1:500
PARP Cell signaling Rabbit 9542S WB 1:400
P53 R&D Mouse MAB1355 WB 1:200
P53 Santa Cruz Mouse sc-126 WB 1:400
Snail Cell signaling Rabbit 3879 WB 1:500
HDAC1 Santa Cruz Mouse sc-81598 WB 1:500
E-cadherin Novus bio Mouse NBP2-19051 WB 1:400
N-cadherin Cell signaling Rabbit 4061 WB 1:200
EGF Receptor Cell signaling Rabbit 2232 WB 1:500
ICAM-1 Santa Cruz Mouse sc-107 WB 1:500
CCL2/MCP-1 Invitrogen Mouse MA5-17040 WB 1:200
Ki67 Vector Laboratories Rabbit VP-RM04 IF 1:100
CD68 Santa Cruz Mouse sc-20060 IF/IHC 1:100

CD3-FITC Biolegend Rat 100204 FACS 1:200
CD3-PE/Cyanine7 Biolegend Rat 100220 FACS 1:200
CD45-APC/Cyanine7 Biolegend Rat 103116 FACS 1:200
CD45-BV510 Biolegend Rat 103138 FACS 1:200
CD8a-PE Biolegend Rat 100708 FACS 1:200
CD4-APC/Cyanine7 Biolegend Rat 100414 FACS 1:200
CD11b-FITC Biolegend Rat 101206 FACS 1:200
CD11b-BV711 Biolegend Rat 101242 FACS 1:200
NK1.1-FITC Biolegend Mouse 108706 FACS 1:200
F4/80-PE Biolegend Rat 123110 FACS 1:200
Ly6G-PE/Cyanine7 Biolegend Rat 127618 FACS 1:200
Ly6C-APC/Cyanine7 Biolegend Rat 128026 FACS 1:200
CD11c-PE/Cyanine7 Biolegend Rat 117318 FACS 1:200
I-A/I-E (MHCll) -APC/Cyanine7 Biolegend Rat 107628 FACS 1:200
CD274(PD-L1)-PE/Cyanine7 Biolegend Rat 124313 FACS 1:200
Siglec F(CD170)-APC Biolegend Rat 155508 FACS 1:200
CD45-PE eBioscience Rat 2-0451-82 FACS 1:200
CD4-PE/Cyanine7 eBioscience Rat 25-0041-82 FACS 1:200
FOXP3-PE eBioscience Rat 12-5773-82 FACS 1:100
CD25-PE/Cyanine7 eBioscience Rat 25-0251-82 FACS 1:200
Arginase-1-PE eBioscience Rat 12-3697-82 FACS 1:100

Validation

UBR5(NB100-1591); EDD (sc-9562); GAPDH (sc-25778), Ki67(VP-RM04), E-cadherin(NBP2-19051): Liao, L. et al. E3 Ubiquitin Ligase UBR5 Drives the Growth and Metastasis of Triple-Negative Breast Cancer. Cancer research, 2017
β-Catenin (9562): Kasumi Murai, et. al.Epidermal Tissue Adapts to Restrain Progenitors Carrying Clonal p53 Mutations. Cell Stem Cell, 2018
Cytokeratin18 (sc-51582): Alisa, A et.al.SIX1 Oncoprotein as a Biomarker in a Model of Hormonal Carcinogenesis and in Human Endometrial Cancer. Mol Cancer Res, 2016
Vimentin (sc-6260): Hae-Yun Jung et.al. Apical-basal polarity inhibits epithelial- mesenchymal transition and tumour metastasis by PAR-complex-mediated SNAI1 degradation. Nat Cell Biol.2019
ZEB1(NBP1-05987): Caterina Miro et.al. Thyroid hormone induces progression and invasiveness of squamous cell carcinomas by promoting a ZEB-1/E-cadherin switch. Nat Commun.2019
ZEB2(sc-48789): L Bakiri et.al.Fra-1/AP-1 induces EMT in mammary epithelial cells by modulating Zeb1/2 and TGFβ expression. Cell Death Differ. 2015
P53 (sc-126): Sun M. et.al. Targeting the Chromosomal Passenger Complex Subunit INCENP Induces Polyploidization, Apoptosis, and Senescence in Neuroblastoma. Cancer research, 2019
PARP(9542S): Jin Yong Kim, et.al. Priming mobilization of hair follicle stem cells triggers permanent loss of regeneration after alkylating chemotherapy. Nat Commun.2019
Snail (3879): Mayra Paolillo, et. al. Stem-Like Cancer Cells in a Dynamic 3D Culture System: A Model to Study Metastatic Cell Adhesion and Anti-Cancer Drugs. Cell, 2019
HDAC1(sc-81598): Lior Soday, et.al. Quantitative Temporal Proteomic Analysis of Vaccinia Virus Infection Reveals Regulation of Histone Deacetylases by an Interferon Antagonist. Cell Rep.2019
N-cadherin (4061): Jason P et.al. Synthetic Lethality of PARP Inhibitors in Combination with MYC Blockade Is Independent of BRCA Status in Triple-Negative Breast Cancer. Cancer research.2018
EGFR (2232), ICAM-1 (sc-107), CD68 (sc-20060): Yin, M. et al. Tumor-associated macrophages drive spheroid formation during early transcoelomic metastasis of ovarian cancer. The Journal of clinical investigation. 2016
CD3-FITC, CD45-APC/Cyanine7, CD8a-PE, CD11b-FITC, NK1.1-FITC, F4/80-PE, Gr-1-PE, CD11c-PE/Cyanine7, MHCll-APC/Cyanine7, CD274(PD-L1)-PE/Cyanine7, CD45-PE, CD4-APC/Cyanine7 CD4-PE/Cyanine7, FOXP3-PE, CD25-PE/Cyanine7, Arginase-1-PE: Song M. et.al. Targeting ubiquitin protein ligase E3 component N-recognin 5 in cancer cells induces a CD8+ T cell mediated immune response. Oncoimmunology. 2020
CD170 (Siglec F)：Jennifer L et.al, Increased flux through the mevalonate pathway mediates fibrotic repair without injury. J Clin Invest. 2019
Dilutions for other antibodies were made according to the manufacturer recommendations.

Eukaryotic cell lines

Policy information about cell lines

Cell line source(s)	ID8 cells, SKOV3 cells, and OVCAR3 cells were obtained from ATCC; Human ovarian surface epithelial cells （HOSEpiC） were purchased from ScienCell Research Laboratories.
Authentication	All cell lines were authenticated by the suppliers before we used them.ID8 ,SKOV3 and OVCAR3 cells were validated by karyotyping .
Mycoplasma contamination	All cell lines were routinely checked for mycoplasma contamination using the MycoAlert detection kit(Lonza). All tests were negative.
Commonly misidentified lines (See ICLAC register)	No commonly misidentified cell lines were used.

Animals and other organisms

Policy information about studies involving animals; ARRIVE guidelines recommended for reporting animal research

Laboratory animals	mouse, C57BL6 strain, female 6-8 week old or 8-12 week old; Rag2-/- (B6 (Cg)-Rag2tm1.1Cgn/J) female 6-8 week old; CD8−/− (B6.129S2-Cd8atm1Mak/J), female 6-8 week old; CD4−/− (B6.129S2-Cd4atm1Mak/J) female 6-8 week old; SCID-Beige female 6-8 week old.
Wild animals	No wild animals were used in this study.
Field-collected samples	No field collected samples were used in the study.
Ethics oversight	All animal experiments were performed in accordance with National Institutes of Health guidelines for housing and care of laboratory animals following the protocol (protocol Number 0701-569A) approved by IACUC at Weill Cornell Medicine.

Note that full information on the approval of the study protocol must also be provided in the manuscript.

Human research participants

Policy information about studies involving human research participants

Population characteristics	Epithelial ovarian cancer (EOC) samples from individuals were obtained from patients aged from 28 to 72 who underwent cytoreductive surgery between 2015 and 2018.
Recruitment	Samples with pathological examination which were confirmed the presence of epithelial ovarian cancer (EOC) were collected.
Ethics oversight	All clinical samples were anonymously coded, and the protocol was approved by the committee for Ethical Review Board at the Comprehensive Cancer Centre of Drum Tower Hospital (Nanjing, China).

Note that full information on the approval of the study protocol must also be provided in the manuscript.

Flow Cytometry

Plots

Confirm that:

- [x] The axis labels state the marker and fluorochrome used (e.g. CD4-FITC).
- [x] The axis scales are clearly visible. Include numbers along axes only for bottom left plot of group (a 'group' is an analysis of identical markers).
- [x] All plots are contour plots with outliers or pseudocolor plots.
- [x] A numerical value for number of cells or percentage (with statistics) is provided.

Methodology

Sample preparation	Peritoneal cells were retrieved from tumor bearing mice and blocking with CD16/32 Fc block after red blood cell lysing, with subsequent antibody staining.
Instrument	FACSCalibur (BC Biosciences)
Software	Flow Jo7.6.1 were used to analyze the data.
Cell population abundance	A minimum of 10,000 post-staining cells were analyzed for each condition.
Gating strategy	Live cells were gated based on FSC/SSC and Zombie UV Fixable viability dye (negative population). Tumor cells and immune subsets were gated with phenotypic gating criteria.

- [x] Tick this box to confirm that a figure exemplifying the gating strategy is provided in the Supplementary Information.
